# Development of surfactin-based nanocarrier for targeted doxorubicin delivery

**DOI:** 10.1038/s41598-026-54757-w

**Published:** 2026-06-13

**Authors:** Omnia Mahareek, Nahla O. Mousa, Samah Mamdouh, Emad M. El-Zayat, Hend Okasha

**Affiliations:** 1 Department of Biochemistry and Molecular Biology, Theodor Bilharz Research Institute, Giza, Egypt; 2 Department of Biotechnology, Faculty of Science, Cairo University, Giza, Egypt

**Keywords:** Surfactin, Lipopeptide, Antibacterial, Anticancer, *Bacillus subtilis*, Biochemistry, Biological techniques, Biotechnology, Cancer, Drug discovery, Microbiology, Nanoscience and technology

## Abstract

Surfactin, one of the most powerful lipopeptide biosurfactants produced by Bacillus subtilis, has great potential for biomedical applications. Isolation, purification, and characterization of surfactin from *Bacillus subtilis* 6633 to be used as a doxorubicin (Dox) nanocarrier. Purified surfactin using different chromatographic columns was used to prepare a self-assembled nanocarrier for Dox, which was characterized for size, charge, and drug-loading efficiency. Biological activity against normal fibroblast (FB) and liver cancer (HepG2) cells was assessed. The hybrid nanoparticles showed a remarkable drug loading; 36%, a pH-responsive release profile with enhanced cytotoxicity toward HepG2 cells (IC₅₀ = 5.40 µg/mL), and a reduced toxicity to FB cells (IC₅₀ = 12.78 µg/mL). The nanoparticles were spherical in shape (100 ± 2 nm) with a polydisperse index of (0.019 ± 0.01) and a narrow size distribution pattern. The results support the potential of surfactin-based nanoparticles to act as a selective platform for anticancer drug delivery and underscore their relevance towards the Sustainable Development Goal 3 (Good Health and Well-being) through the development of safer and effective therapeutic strategies.

## Introduction

Biosurfactants can be of various chemical structures, including fatty acids, glycolipids, lipopeptides, lipopolysaccharides, and lipoproteins, depending on the generative microorganism, raw materials, and processing conditions. Lipopeptides (LPs) are among the best-known subclasses of biosurfactants (biological surfactants). Market growth has been driven by consumer awareness and demand for sustainable and biodegradable products. LPs are surface-active compounds produced by bacteria, fungi, and cyanobacteria in nature and have a variety of structural and functional properties. These secondary metabolites are bioactive and have considerable medicinal and biotechnological implications. Non-ribosomal peptide synthases have been the source of the bulk of the LPs that have been identified since the 1940 s^1,2^. Due to the related risks to the environment and public health, synthetic surfactants should be replaced by biosurfactants as environmentally benign dispersants. Compared to synthetic surfactants, biosurfactants have a number of benefits, such as lower toxicity, increased biodegradability, environmental compatibility, tolerance to high temperatures, and a broad spectrum of biological activities^1,3^. One of the most potent biosurfactants known to date is surfactin, a cyclic lipopeptide produced by several strains of Bacillus subtilis. It has hydrophilic and lipophilic moieties. The hydrophilic group has carboxyl, phosphoryl or hydroxyl functionalities contributed by fatty acids, amino acids, phospholipids, saccharides or peptides while the lipophilic group is a hydrocarbon chain of a fatty acid or sterol ring. Structurally, surfactin is a 13–16 carbon fatty acid chain linked to a heptapeptide (L-Glu-L-Leu-D-Leu-L-Val-L-Asp-D-Leu-L-Leu). Many homologs are produced by variations at positions 2, 4, and 7 of the heptapeptide. Surfactin has a wide range of biological activity against both Gram-positive and Gram-negative bacteria and fungi, such as antiviral, antiprotozoal, anti-mycoplasma, and broad-spectrum antimicrobial properties^4,5^. In order to achieve maximum lipopeptide yield, the influence of fermentation parameters on yield and composition should be investigated. It has been reported in the literature that the composition of the growth medium and fermentation conditions have a strong influence on the optimum production of antimicrobial lipopeptides. This present study was conducted to (i) molecularly confirm the surfactin gene in *B. subtilis* 6633, (ii) optimize its production in different culture conditions, (iii) isolate and characterize surfactin, and (iv) evaluate its biological activity and potential as a nanocarrier for doxorubicin. Doxorubicin is an anthracycline anticancer drug, used in the treatment of different types of tumors like breast cancer, leukemia, liver, lymphoma, sarcoma, ovarian, bladder, and lung cancers due to its strong interaction with DNA and topoisomerase II inhibition. However, its application is limited by the severe side effects, including cardiotoxicity and the development of multidrug resistance (MDR) in cancer cells. Nanocarrier-mediated delivery approaches have been proposed to improve the selectivity of doxorubicin for the tumor and to decrease its side effects. The distribution of doxorubicin has been extensively studied in various drug carrier systems, including liposomes, polymeric nanoparticles, and micelles, which exhibit improved pharmacokinetic properties and lower toxicity^6–8^.

Because of their biocompatibility and wide range of applications, biosurfactant-based systems have recently gained popularity. Although surfactin, a cyclic lipopeptide released by *Bacillus subtilis*, has already demonstrated its usefulness in drug loading and nanostructure construction, little research has been done on creating surfactin-based nanosystems for doxorubicin delivery^9,10^. Notably, surfactin-based nanoparticles have been successfully utilized for doxorubicin delivery. Huang et al^11^. reported the development of surfactin nanoparticles (DOX@SUR), which significantly enhanced the cellular uptake and overcame the multidrug resistance in cancer cells with improved anticancer activity compared with free doxorubicin. To the best of our knowledge, no other research has studied the delivery of doxorubicin using the combined surfactin-chitosan system. In this context, the present work is dedicated to the design and characterization of a new hybrid nanocarrier based on chitosan and surfactin from Bacillus subtilis 6633. The proposed system is intended to improve the properties of nanoparticles, increase the drug loading capacity, and to ensure pH-responsive drug release.

The developed nanocarriers were also tested in vitro on cancer and normal cell lines to evaluate their toxicity and their ability to enhance the anticancer effects of doxorubicin, while exerting less effect on normal cells. The dual assessment allows a more comprehensive assessment of the efficacy and safety of the therapy.

## Materials and methods

### Confirmation of srf gene presence using PCR

The *Surf* 176 bp fragment, corresponding to the *B. subtilis sfp* gene (GenBank accession no CP034943.1) at positions 384,088–384,264, was PCR-amplified using the following primers (Biovision, Inc, USA), sense: GGGCTTTGATCCTGAACGG-3, and antisense: ATCATCTTTCGCTTGGCCCT. Amplification was accomplished with BIO-RAD T100 Thermal Cycler according to^4^. Different annealing temperatures (56 to 62 °C). After the amplification, the PCR reaction product was analyzed by 2% agarose gel electrophoresis.

### Studying culturing conditions on *Bacillussubtilis* 6633

Ten-fold dilutions of *Bacillus* subtilis ATCC6633 (Central Reference Laboratory for Public Hospitals) were prepared and cultivated on four LB agar plates incubated at 37 °C for 24 h.

Mineral salt medium (MSM) was first prepared according to^12^ with a modification^13^ whereas 2% of filtered glucose was added to the medium. Three colonies of (1:100,000) were inoculated into 50 mL of MSM medium and incubated at 150 rpm, 35 ^◦^ C, for 72 h^14,15^.

### Optimization of fermentation media for surfactin production

#### Carbon and nitrogen sources

Carbon sources used were: glucose, glycerol, maltose, sucrose, dextrose, and used cooking oil. MSM was prepared with a stable nitrogen source concentration (0.4% ammonium sulfate) and tested carbon source concentrations ranging from 0.1% to 4% depending on the source. *B. subtilis* was cultivated for three days^16,17^. For nitrogen source, four concentrations of ammonium sulfate were selected: 0.2, 0.4, 0.8, and 1.6%, in MSM media containing 2% gluose^18,20^.

#### Effect of pH and temperature

Four pH values were selected: 5.7, 6.7, 7.7, and 8. MSM was prepared by adding 2% glucose. For studying the temperature effect, five temperatures were selected: 31, 33, 35, 37, and 39 °C. The cultured bacteria were incubated for three days^19,20^.

### Extraction and purification of surfactin

The crude biosurfactant was initially recovered by acid precipitation followed by solvent extraction, in which the cell-free supernatant was obtained by centrifugation of the culture broth at 5,000 rpm and 4 °C for 40 min. According to Barale et al., acid precipitation was carried out using 1 M HCl to obtain a pH of 2. The culture supernatant was then incubated for 24 h at 4 °C followed by centrifugation at 10,000 rpm and 4 °C for 40 min, then extraction using 70% ethanol (v/v), evaporated at 40 °C, and dried at 40 °C. The surfactin yield was determined gravimetrically by weighing the dried extract, as previously described for surfactin production in *Bacillus subtilis*. All measurements were performed in triplicate^21,22^.

### Purification of surfactin using FPLC

AKTA Purifier 100 FPLC system (GE Healthcare Life Sciences, Sweden) was used in the purification of surfactin using different column types.

#### Desalting chromatography

The partially purified bacterial lipopeptide pellet, surfactin, with a concentration of 0.673 mg/mL, was applied onto 5 mL HiTrap column (GE Healthcare Life Sciences) and eluted with sodium phosphate buffer (20 mM; pH 7.5) with 5 column volumes (CV). Fractions were collected for further assessment. chromatography for lipopeptide, surfactin, purification^21,23^.

#### Anion exchange chromatography

Weak anion DEAE (Diethylaminoethyl) cellulose, anion ANX, and Hitrap QFF 1 mL columns (GE Healthcare Life Sciences) were utilized and equilibrated with washing buffer (20 mM Tris buffer pH 6.8) and elution buffer (20 mM Tris pH 6.8 and 1 M NaCl). A 0.2 mg of purified surfactin from desalting was loaded. The column was washed using the washing buffer to remove other proteins, then linear gradient elution (20 CV) was carried out using the elution buffer. The eluted peaks were collected, and oil displacement activity was evaluated. The active fractions were further analyzed by HPLC, FTIR, NMR, and GC–MS^47^.

### Oil displacement assay

The fractions obtained were assessed for oil-displacement activity. In a Petri dish with a diameter of 8 cm, 30 mL of distilled water was placed. Subsequently, 200 µL of crude oil was used to form an oil film under the aqueous surface, then 10 µL of a surfactin solution was added under the oil surface. Oil dispersion was measured by the diameter of the halo formed using ImageJ software. Anionic surfactant sodium dodecyl sulfate (SDS) was used as a positive control, and water was used as a negative control^24^.

### Thin-layer chromatography (TLC)

The fractions were subjected to TLC using Silica-coated aluminum plate immersed in chloroform: methanol: acetic acid (85:10:5). The plate was developed, dried, and visualized under UV light of 254 nm and 365 nm (Silufol, Kavalier Germany) for fluorescence quenching spots, and Rf value of the surfactin spot was evaluated using the following formula and results recorded^25^.

$${\mathrm{Rf}} = \;{\text{Distance travelled by the solute (cm)/Distance travelled by the solvent (cm)}}.$$

### Characterization of surfactin

#### High Performance Liquid Chromatography (HPLC)

The purified lipopeptide was injected to HPLC chromatograph YL-9100 system with a C18 column (Agilent Technologies). At a flow rate of 1.0 mL/min, a gradient elution was performed over 35 min using buffer A (0.1% TFA in water) and buffer B (0.1% TFA in acetonitrile)^26^.

#### Infrared analysis (FTIR)

The samples were prepared by mixing 1 mg of purified surfactin with 100 mg of KBr and pressing the mixture into a pellet form at 134 MPa for 2–3 min to obtain transparent pellets. The IR spectrum of the pellet was collected from 400 to 4000 wavenumbers (cm^− 1^), and is an average of 32 scans obtained using a FTIR (4100typeA) spectrometer^27^.

#### Hydrogen and carbon nuclear magnetic resonance (NMR)

A 400 MHz spectrometer was used to perform the H NMR of the purified surfactin. The material was dissolved in deuterated methanol (MeOD) at 298.1 K to obtain the spectra. A 100 MHz spectrometer was used for the C NMR analysis, and the sample was prepared in deuterated chloroform (CDCl₃). To achieve the best resolution, both spectra were processed using typical exponential window functions (LB = 0.30 Hz for H)^28^.

#### GC-mass analysis of beta-hydroxy fatty acids in surfactin

GC-MS operating analysis was performed using Thermo Scientific TRACE GC Ultra system (Thermo Fisher Scientific, Walthman, MA UAS). TSQ8000 evo triple quadrupole model specification equipment and injector TR-59MS USA equipment with a 30-mm diameter, 0.25-mm internal diameter, and 0.25-mm film thickness was used. Temperatures for the injector and detector was 270. The opening oven temperature was held for five minutes at 50 °C and then allowed to increase to 230 °C at a rate of 5 °C/min. The flow rate for the helium gas was fixed at 1 mL/min. Prior to injection, the sample was diluted at a ratio of 1:10 in petroleum ether and purified using a 0.2 L m filter with syringe attachment. The collected components were matched with NIST database equipped with GC-MS method to classify the sample’s chemical compounds^29^.

### Preparation of the Dox-loaded surfactin/chitosan nanoparticles

Dox-Surf/CS nanoparticles were produced using a micelle-assisted ionic gelation technique as described by^11,30^. A0.5 mL of Dox.HCl solution (1 mg/mL) and 0.5 mL of surfactin solution (2.5 mg/mL) were combined. To create a sustain dispersion, the mixture was gently vortexed every 10 min for 30 to 60 min at room temperature (25 °C). the Dox–surf micelles (with a mass ratio of 1:2.5 and total volume 1 mL) was firstly centrifuged to collect the generated nanoparticles at 15,000 rpm for 30 min at 10 °C using an ultracentrifuge (supra25K, Hanil science industrial, Korea) and the supernatant was decanted and stored. The pellet was resuspended in 1 mL deionized H_2_O and gradually added to the 10 mL 0.2% chitosan solution in 1% acetic acid. After 15 min of stirring, 4 mL of sodium tripolyphosphate (TPP) solution (1 mg/mL in deionized water) was gradually added over the course of 5–10 min to initiate ionic crosslinking between the cationic chitosan and the anionic TPP. To ensure full gelation and stability of the Dox-Surf/CS nanoparticles, stirring was maintained for a further half hour to an hour. Centrifugation at 15,000 rpm for 10 min at 10 °C was performed, and The pellet was resuspended in 1 mL of deionized water and directly subjected to the freeze dryer (Edwards Modulyo, UK) to produce dried powder, which was then collected and kept at 4 °C. The Dox/CS without Surf and the blank CS nanoparticles without both Dox and Surf were formed by the same method.

#### Evaluation of Dox encapsulation efficiency and drug loading

The supernatant obtained from Dox-Surf/CS nanoparticles was used to indirectly quantify the drug loading content (DL%) and encapsulation efficiency percentage (EE%). A UV-Vis spectrophotometer (Model: se6100 UV-Vis double beam, Abbotta Corporation, USA) was used to measure the sample three times at 480 nm^11,31–33^. The data were displayed as the mean ± SD. Using known Dox.HCl values (1, 0.5, 0.25, 0.125, 0.125, 0.0625, 0.03125, 0.0156, 0.0078, 0.0039, 0.00195, 0.000975, and 0.0000487) mg/mL, a standard calibration curve, and equations (1) and (2) were used to determine the EE% and DL%.

1 $$\:\:\:\:\:\:\:\:\:\:\:\:\:\:\:\:\:\:\:\:EE\left(\mathrm{\%}\right)=\frac{\:CONC\:administered\:Dox-CONC\:of\:free\:Dox}{conc\:of\:total\:Dox}100$$

2 $$\:DL\left(\%\right)=\frac{M\:administered\:Dox-M\:free\:Dox\:\:\:}{M\:of\:surf}\times\:100$$

where CONC of administered Dox, M of administered Dox, and M of Surf are the initial concentrations. M free Dox and M of surf: are the mass of Dox and Surfactin used in the nanoparticle formation technique.

#### In vitro release study of Dox from both the DOX-Surf/CS and DOX- CS nanoparticles

The released amount of Dox from the Dox-Surf/CS and Dox/CS nanoparticles was investigated at two pH values in simulated dissolution media: pH 4.8 simulating the pH of cancerous cells, and pH 7.4 simulating the environment of the normal tissues, over a period of 72 h. A 1 mg sample of Dox-Surf/CS and Dox- CS nanoparticles was separately added to 2 mL of 1x PBS at (pH 7.4 or pH 4.8) and incubated shaking at 37 °C. A 200-µL aliquot of the supernatant was taken at different time intervals (0.5, 1, 2, 4, 6, 12, 24, 48, and 72 h) and supplemented with a 200-µL fresh 1x PBS solution to maintain the total volume. The absorbance of released Dox was measured at 480 nm using UV–Vis spectrophotometer according to Fahmi et al., standard curves^31^.

$$\:The\:cumulative\:drug\:release\:\left(\%\right)=\:\frac{amount\:of\:Dox\:released}{initial\:amount\:of\:Dox}x\:100\:\:\:\:\:\:\:\:\:\:\:\:\:\:\:\:\:\:\:\:\:\:\:\:\:\:\:\:\:\:\:\:\:\:\:\:\:\:\:\:\:\:\:\:\:\:\:\:\:\:\:\:\:\:$$

#### Morphology of Dox‑Surf/CS nanoparticles

The shape of the nanoparticles was determined using a transmission electron microscope (TEM) (JEM-1400, Jeol, USA). The average size and PDI of the nanoparticles were measured using DLS (Malvern, UK). The sample was diluted and sonicated for 5 min and then measured at room temperature. The surface charges of the obtained nanoparticles were measured as a function of zeta potential by DLS (Nanotrac, wave2, UK).

### In vitro studies of Dox-Surf/CS nanoparticles

#### Hemolytic activity assay

Blood was collected from three adult healthy male mice (Swiss albino, weighing 25 ± 2.6 g) via the retro-orbital sinus under light anesthesia, and whole blood was collected in EDTA tubes. The animals were obtained from the animal facility of TBRI. All experimental procedures involving animals were conducted in accordance with relevant guidelines and regulations (Approval No: FWA 00010609/PT 766).

The hemolysis assay was performed according to Mohamed et al., Serial dilutions of surfactin in PBS from 170.66 to 0.33 µg/mL were prepared from acid-precipitated and solvent-extracted fractions. RBCs with PBS (no surfactin) served as the negative control, and RBCs mixed with 0.01% Triton X-100 served as the positive control. The hemolysis percentage was calculated using the following equation, and the data were displayed as the mean ± SD^13,34^.

$$\% {\mathrm{Hemolysis}}\: = \frac{{({\mathrm{OD}}540\;{\mathrm{sample}}\: - \:\:\:{\mathrm{OD}}540\:{\mathrm{negative}}\:{\mathrm{control}})}}{{({\mathrm{OD}}540\:{\mathrm{positive}}\:{\mathrm{control}} - \:\:\:{\mathrm{OD}}540\:{\mathrm{negative}}\:{\mathrm{control}})}} \times \:100$$

#### In vitro cytotoxicity on normal and cancer cell lines

Normal fibroblast (FB) and liver cancer (HepG2) cell lines (Department of Cell Culture, Vacsera, Egypt) were chosen by the crystal violet assay^35^. The HepG2 and FB cells were cultured separately in two 96-well cell culture plates at a density of 5000 cells per well for 24 h at 37 °C in a 5% CO2 incubator in a DEMEM media containing 10% Fetal Bovine Serum (FBS) (Lonza Bioscience), 1% antibiotic Fungizone (Lonza Bioscience), and 1% HEPES (BioWest, USA). Cells were then treated for 24 h with free Dox, Dox-Surf/CS nanoparticles, and Dox-CS nanoparticles solutions, separately at concentrations suspended in a DEMEM media containing 2% FBS. In parallel, he cytotoxic effect of empty chitosan nanoparticles and surfactin solution (1000, 500, 250, 125, 62.5, 31.25, 15.625, 7.8, 3.9, 1.9, and 0) µg/mL was also studied against HepG2 and FB cell lines. After crystal violet staining, the absorbance at 570 nm was measured using a microplate reader, and cell viability is expressed as a percentage of viability using the following equation:

$$\:Viable\:cells\left(\%\right)\:\:=\:\:\frac{\mathrm{A}\:\mathrm{T}\mathrm{r}\mathrm{e}\mathrm{a}\mathrm{t}\mathrm{e}\mathrm{d}\:}{\mathrm{A}\:\mathrm{c}\mathrm{o}\mathrm{n}\mathrm{t}\mathrm{r}\mathrm{o}\mathrm{l}\:\:}\times\:100\:\:\:\:\:\:\:\:\:\:\:\:\:\:\:\:\:\:\:\:\:\:\:\:\:\:\:\:\:\:\:\:\:\:\:\:\:\:\:\:\:\:\:\:\:\:\:\:\:\:$$

Each experiment was carried out in triplicate, and the results are expressed as mean ± SD.

### Statistical analysis

The data were presented as the mean ± SD of at least three replicates. The test of significance was performed by GraphPad Prism 7 (San Diego, California, USA). A two-tailed multiple T-test was employed to determine the significance of differences between normal and cancer cells. The ρ-value < 0.05 was considered to indicate a statistically significant difference.

## Results

### Confirmation of srf gene presence using PCR

**Fig. 1:**
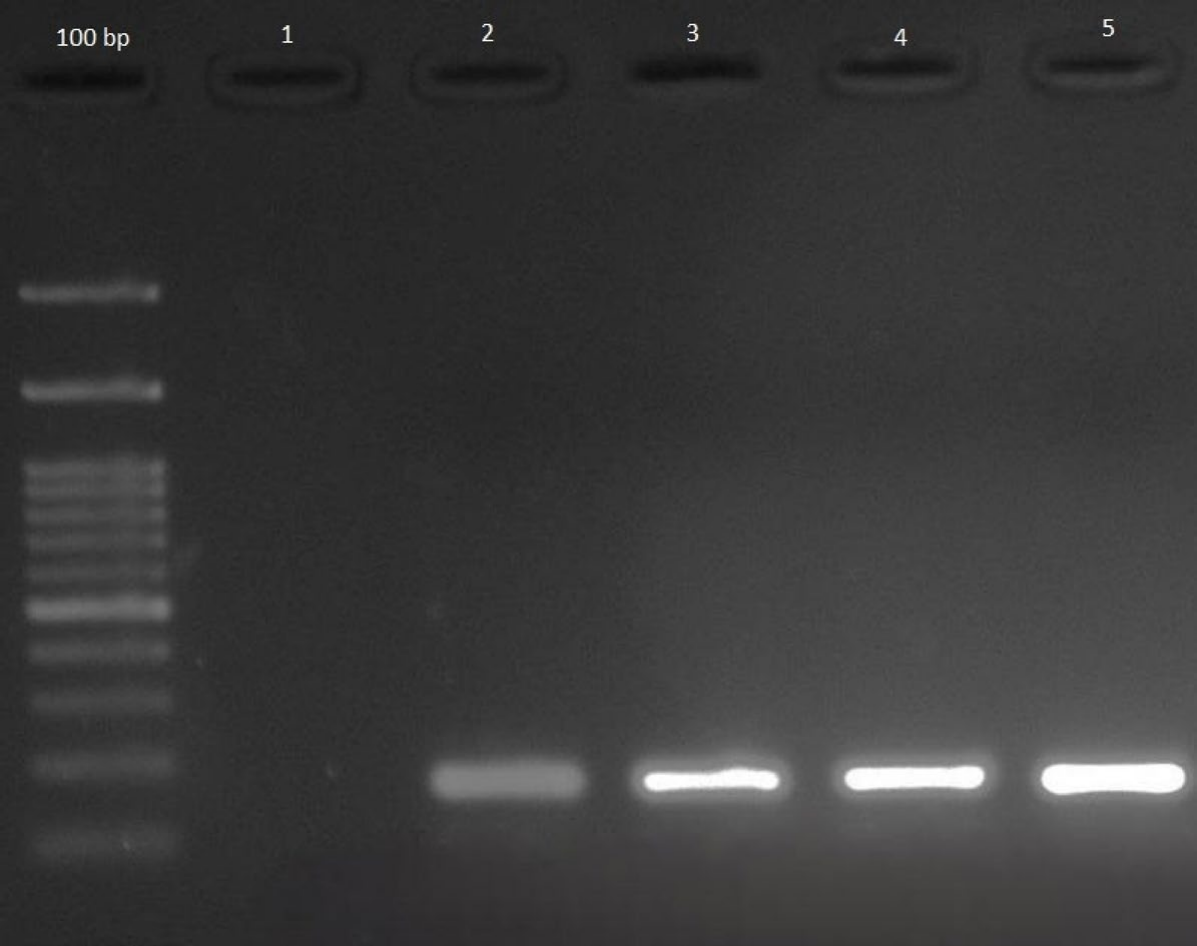
PCR products of the srf gene electrophoresed on a 2% agarose gel. the results of PCR amplification of the Bacillus subtilis 6633 srf gene using Surf-F and Surf-R primers. Negative control is lane 1. Annealing temperatures in lanes 2 through 5 are 56, 58, 60, and 62 °C, respectively. At 62 °C, the highest and most distinctive band was seen. At this temperature, no non-specific bands were seen. The size marker was a 100 bp DNA ladder.

The expected 176 bp segment of the Bacillus subtilis *Surf* gene has been amplified effectively by PCR using primers *Surf* -F and *Surf* -R. Reactions conducted at annealing temperatures (56, 58, 60 and 62) °C, from lane 2 to lane 5, respectively, and lane 1 was the negative control, produced distinct, transparent bands, with maximum intensity amplification occurring at 62 °C as shown in [Fig. 1]. There was no evidence of primer-dimer production or non-specific amplification. These findings verify that the *Surf* gene is present in the genomic DNA of B. subtilis 6633.

### Studying culturing conditions on *Bacillus subtilis* 6633

After 24 h at 35 °C, well-isolated colonies on LB agar were obtained with a 1:100,000 dilution of *B. subtilis* 6633. The colonies were spherical, creamy, and slightly uneven at the borders and used for further inoculation into MSM with an OD reading of more than one after incubation for 72 h.

### Optimization of fermentation media for surfactin production

#### Carbon and nitrogen sources

**Fig. 2:**
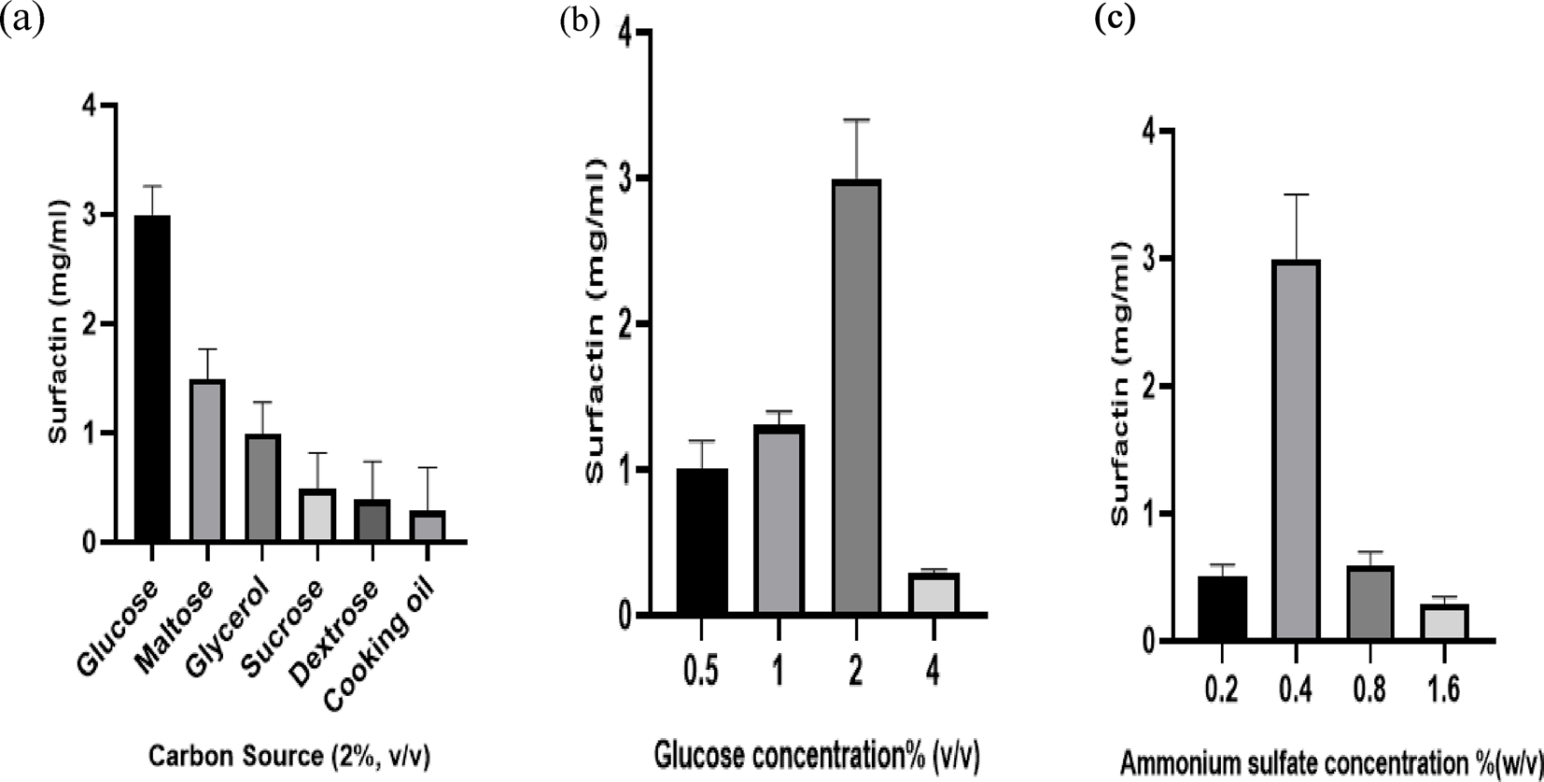
Optimization of surfactin production by Bacillus subtilis 6633 under various nutritional conditions. (**a**) Effect of various carbon sources on surfactin production, where glucose gave the highest yield of surfactin (3 mg/mL). (**b**) Effect of glucose concentrations (%v/v) on surfactin production, where 2% glucose gave the maximum surfactin yield, whereas a higher glucose concentration (4%) gave a lower yield. (**c**) Effect of ammonium sulfate concentrations (%w/v) as a nitrogen source, where the optimal yield was obtained at 0.4%. The results showed that the conditions were significantly different from each other (*p* < 0.0001).

According to the results [Fig. 2-a], glucose produced the highest surfactin concentration (3 mg/mL), followed by maltose (1.5 mg/mL), glycerol (1 mg/mL), sucrose (0.5 mg/mL), dextrose (0.4 mg/mL), and cooking oil (0.3 mg/mL). Statistical analysis revealed that the differences in surfactin production across these carbon sources were highly significant (*p* < 0.0001). By using glucose concentrations of 0.5%, 1%, 2%, and 4% (v/v) in MSM supplemented with 0.4% (w/v) ammonium sulfate and 0.1% salt solution [Fig. 2-b], the highest surfactin yield was obtained at 2% glucose (3 mg/mL), followed by 1% (1.3 mg/mL) and 0.5% (1 mg/mL). Yields dropped sharply at 4% glucose (0.3 mg/mL). Differences across concentrations were highly significant (*p* < 0.0001).

At 0.4% ammonium sulfate, the highest surfactin yield (3 mg/mL) was recorded; yields at 0.2% (0.5 mg/mL) and 0.8% (0.6 mg/mL) were substantially lower. The lowest yield (0.3 mg/mL) was observed at the highest nitrogen concentration (1.6%). These results indicate that 0.4% ammonium sulfate is the optimal concentration for surfactin biosynthesis. Statistical analysis confirmed significant differences in yields across nitrogen concentrations (*p* < 0.0001) [Fig. 2-c].

#### Effect of pH and temperature

**Fig. 3:**
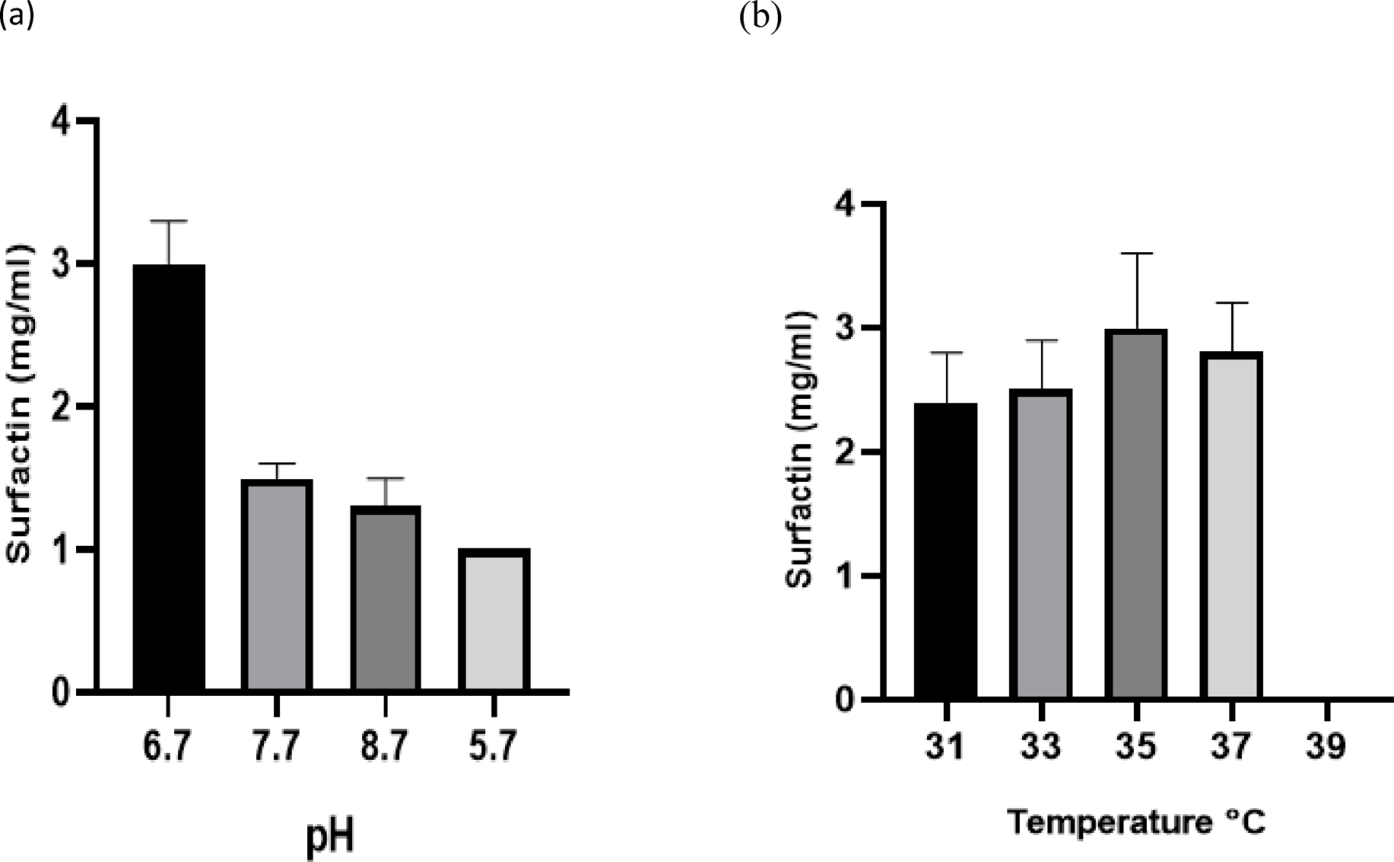
*Bacillus subtilis* 6633’s surfactin synthesis is optimized under different environmental conditions. (**a**) The impact of temperature on the production of surfactin, with no growth at 39 °C and maximum production at 35 °C. (**b**) The impact of pH, where output peaked at pH 6.7 and then decreased at lower pH levels. For every condition, significant differences were found (*p* < 0.0001).

The highest output was recorded at pH 6.7 (3 mg/mL) during pH optimization, followed by pH 7.7 (1.5 mg/mL), pH 8.7 (1.3 mg/mL), and pH 5.7 (1 mg/mL). Differences in surfactin production across pH levels were highly significant (*p* < 0.0002). Temperature optimization revealed that the maximum production occurred at 35 °C, with yields decreasing at temperatures below and above this optimal range, and no growth occurred at 39 °C. Statistical analysis confirmed significant differences in surfactin yield across temperatures tested (*p* < 0.0001). This demonstrates that *B. subtilis* 6633 is temperature-sensitive and that the optimal surfactin synthesis requires careful management of both pH and temperature, as shown in [Fig. 3].

### Purification of surfactin using FPLC

#### Desalting chromatography

**Fig. 4:**
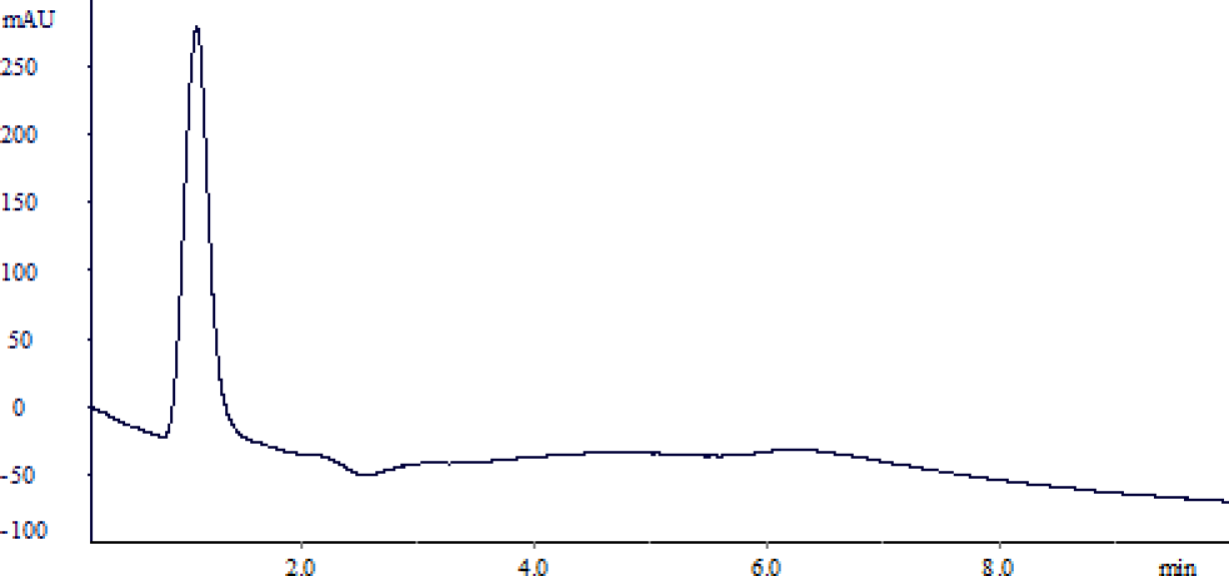
Desalting chromatographic profile of the generated lipopeptide surfactin using a 5 mL pre-equilibrated column filled with 20 mM sodium phosphate buffer (pH 7.5). After around ten minutes, a single peak was seen, signifying that the surfactin purification process had been successful. Surfactin was present in the collected fraction at a value of 0.186 mg/mL.

On a pre-equilibrated 5 mL desalting column with 20 mM sodium phosphate buffer (pH 7.5), a single major peak emerged approximately ten minutes after purification [Fig. 4]. The pooled fraction’s surfactin concentration of 0.186 mg/mL revealed a satisfactory buffer exchange and recovery.

#### Ion exchange chromatography

**Fig. 5:**
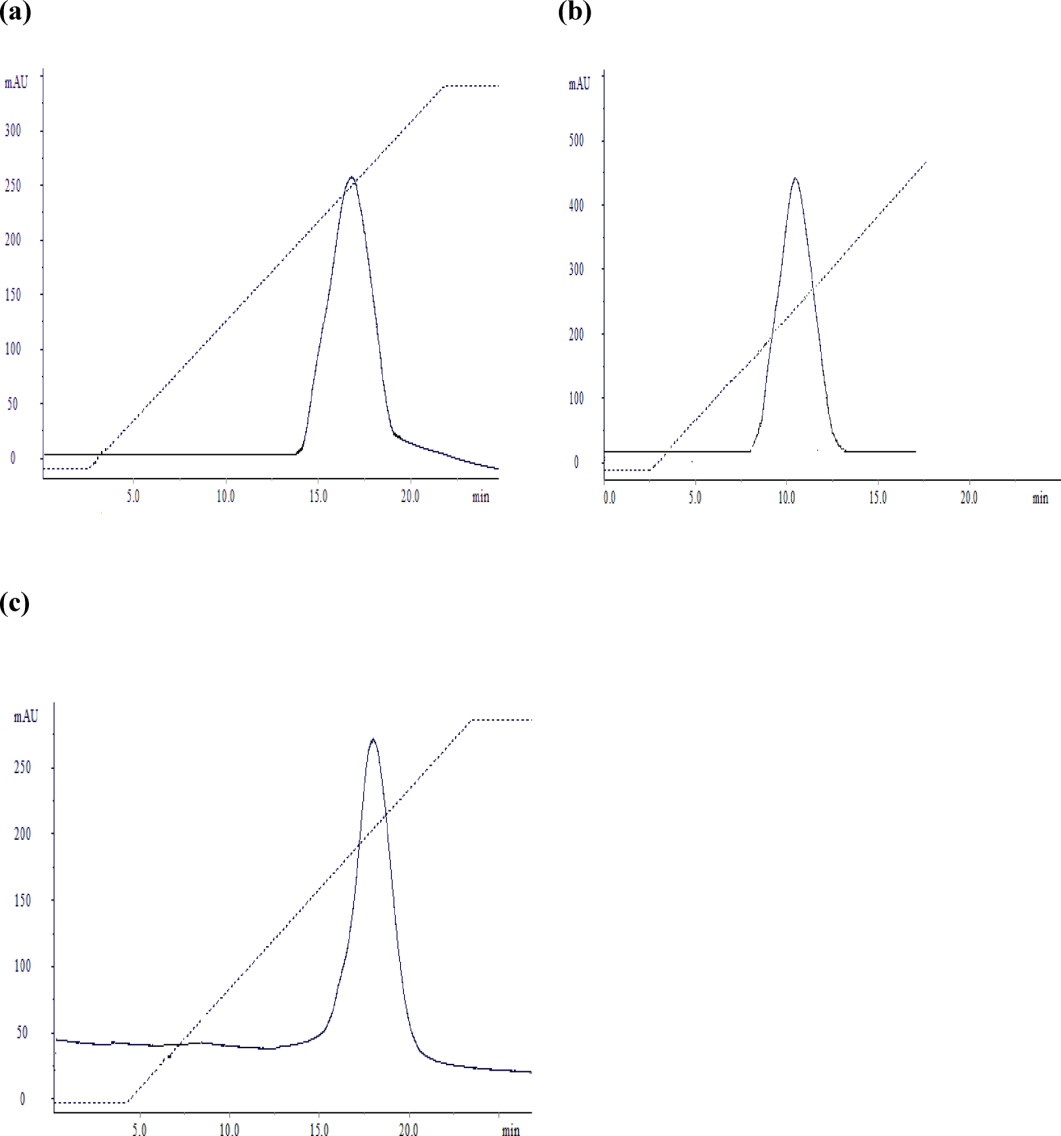
Surfactin is purified by ion exchange chromatography using a variety of anion exchange columns. (**a**) DEAE-cellulose, (**b**) ANX, and (**c**) HiTrap Q Sepharose Fast Flow columns that were eluted using a NaCl gradient after being pre-equilibrated with 20 mM Tris-HCl buffer (pH 6.8). The chromatograms show that all columns have well-resolved surfactin peaks. The y-axis shows the absorbance at 210 nm in mAU, while the x-axis shows the time in minutes.

Different ion exchange chromatography columns were used to purify the antimicrobial lipopeptide, surfactin, from the fraction collected through desalting chromatography using three distinct anion exchange resins. Three consecutive anion exchange columns—DEAE-cellulose, ANX, and HiTrap Q Sepharose Fast Flow—were used to purify surfactin. A NaCl gradient in 20 mM Tris-HCl at pH 6.8 was used to pre-equilibrate and elute these, producing single peaks with increasing resolution [Fig. 5a-c].

### Oil displacement assay

**Fig. 6:**
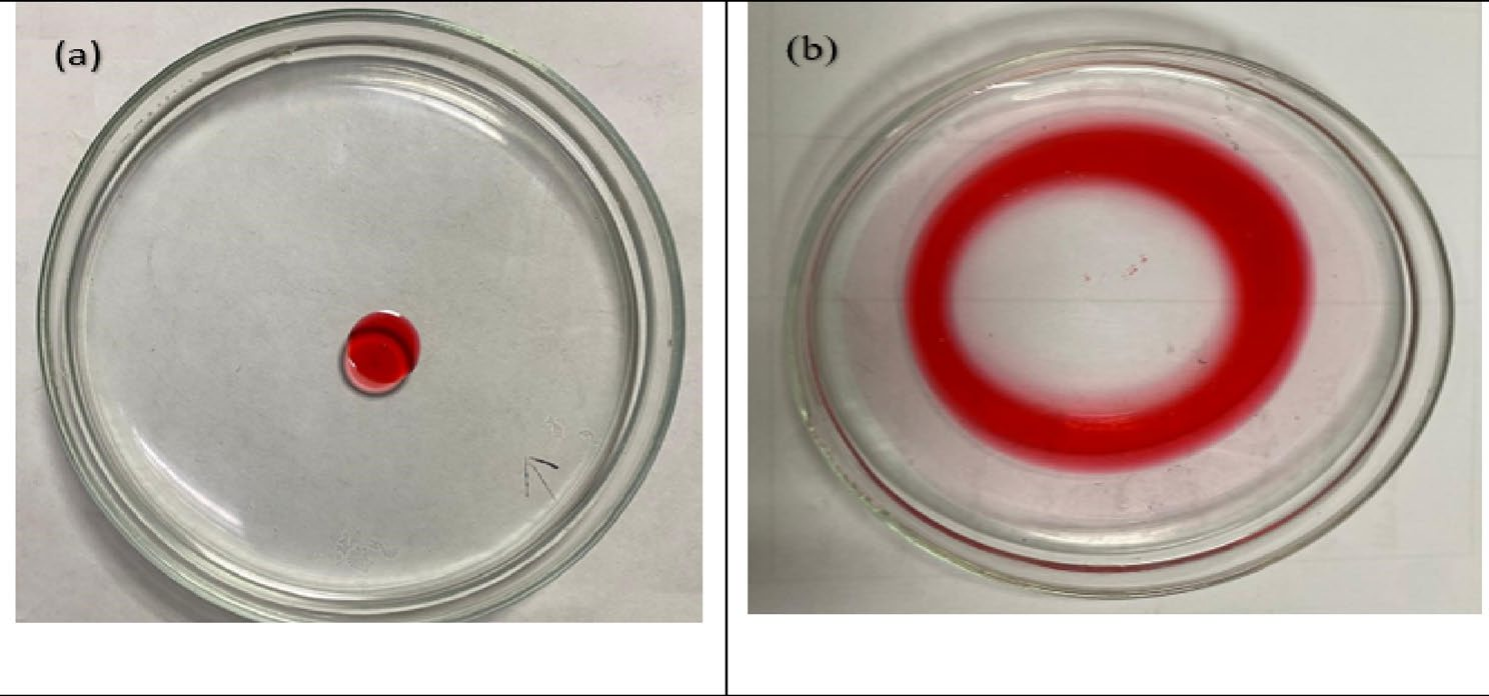
Oil Displacement Assay for Surfactin Surface Activity. (**a**) A clear halo zone that shows how well surfactin displaces oil. (**b**) Negative control, in which oil was not displaced by distilled water. The biosurfactant activity of the generated surfactin is indicated by the existence of a clear zone.

As shown in Fig. 6, the oil displacement assay showed a definite halo zone with a quantifiable diameter [Fig. 6-a] in contrast to the control where dH2O was added [Fig. 6-b].

### Thin-layer chromatography

**Fig. 7:**
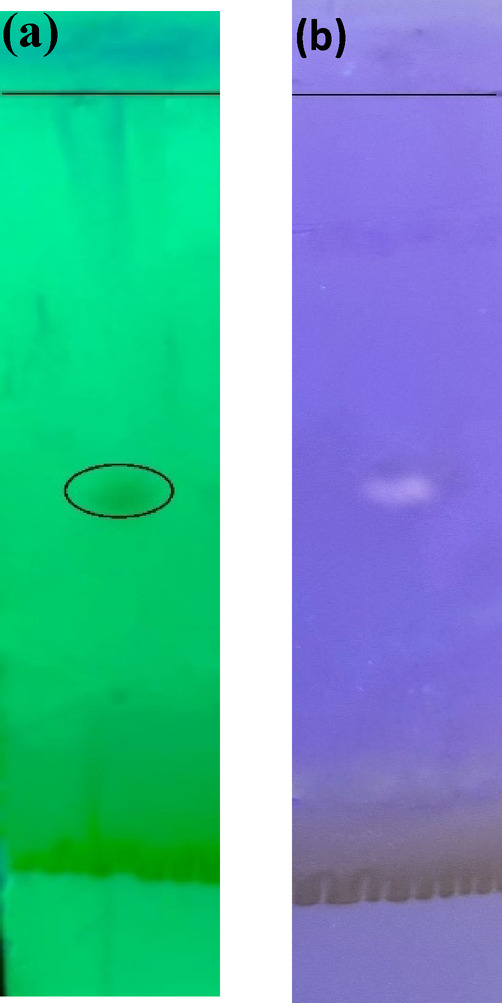
Purified surfactin produced by Bacillus subtilis 6633 using thin-layer chromatography (TLC). Under UV illumination, a clear band with an Rf of 0.54 was seen at (**a**) 254 nm and (**b**) 365 nm, signifying the presence of the lipopeptide surfactin.

Subsequent examination using thin-layer chromatography showed a clear band with an Rf value of 0.54 of the lipopeptide surfactin visualized under UV light of 254 nm [Fig. 7-a] and 365 nm [Fig. 7-b].

### Characterization of surfactin

#### High Performance Liquid Chromatography (HPLC)

**Fig. 8:**
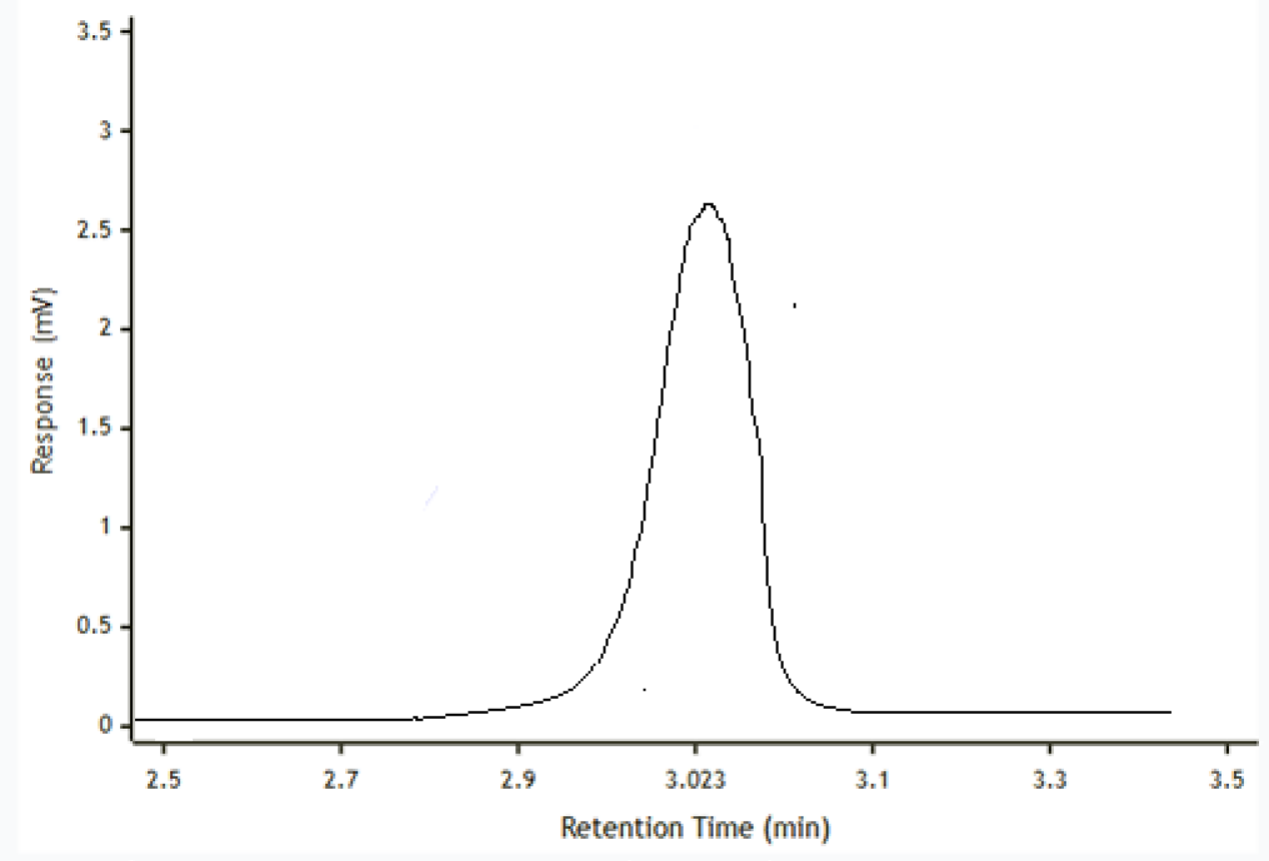
HPLC analysis of Bacillus subtilis 6633 pure surfactin following ultrafiltration. The predominant peak in the chromatogram at a retention time (Rt) of 3.02 min under isocratic conditions shows that the surfactin was successfully purified. The time in minutes is shown on the x-axis, while the detector response in millivolts is shown on the y-axis.

As shown in [Fig. 8], surfactin was extracted from *Bacillus Subtilis* 6633 and purified through the ultrafiltration strategy was further subjected to HPLC analysis under isocratic exhibited a major peak at a retention time (Rt) of 3.02 min.

#### Infrared analysis (FTIR)

**Fig. 9:**
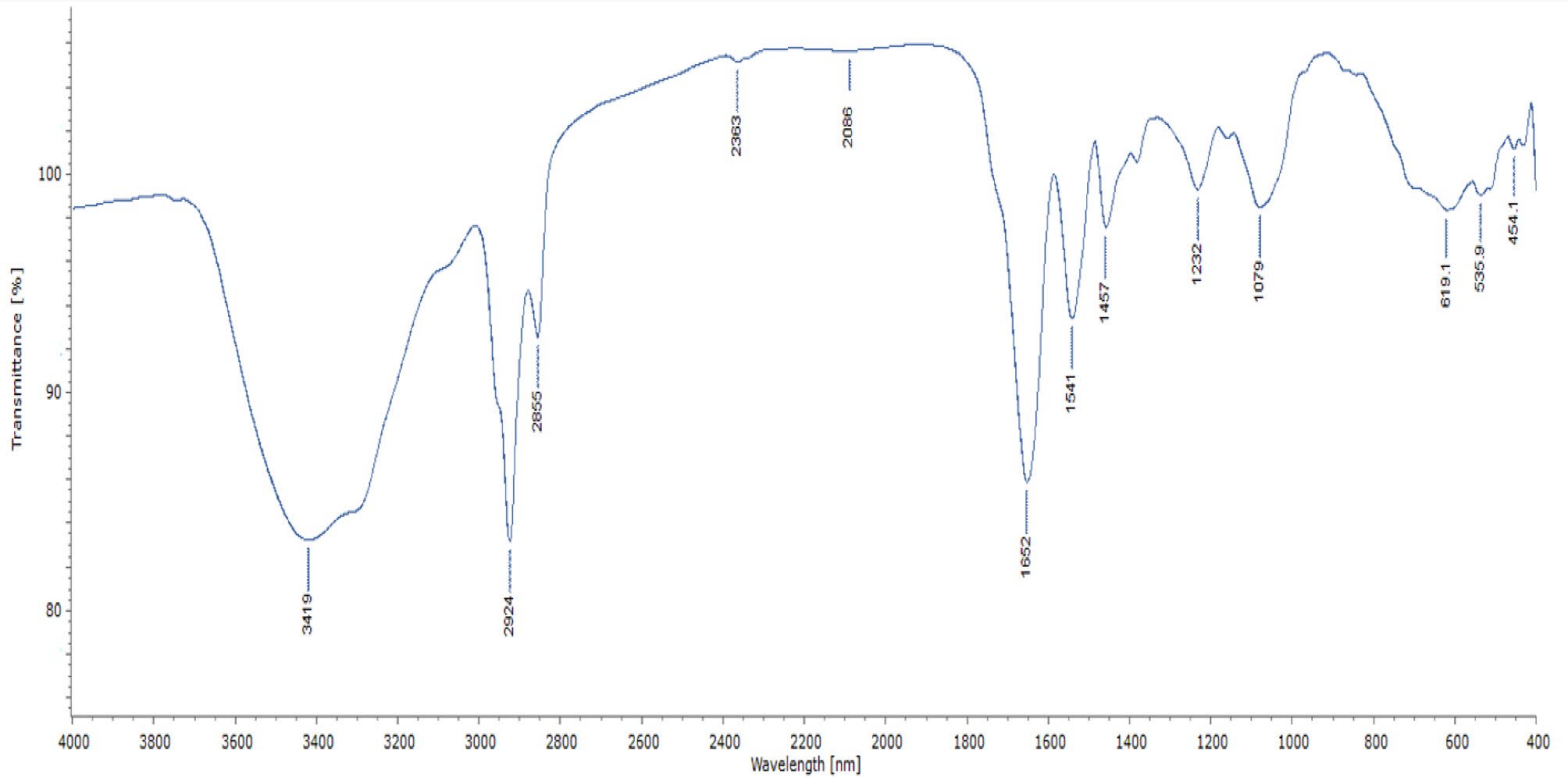
FT-IR spectra of pure surfactin scanned as KBr pellets (400–4000 cm⁻¹). The presence of peptides and aliphatic chains is indicated by the significant absorption peaks for O-H/N-H stretching (3419 cm⁻¹), C-H stretching of aliphatic chains (2970 − 2850 and 1450–1380 cm⁻¹), C = O stretching of peptide bonds (1650 cm⁻¹), and N-H bending and C-N stretching (1540 cm⁻¹). Wavelength is displayed on the x-axis, and transmittance (%) is shown on the y-axis.

As shown in [Fig. 9], the FT-IR spectrum of *B. subtilis* 6633 revealed characteristic functional groups consistent with the presence of surfactin. Strongly absorbing bands, consistent with the presence of a peptide component at 1650 cm − 1 resulting from the stretching mode of the CO–N bond, and at 1540 cm − 1 from the deformation mode of the N–H bond combined with the C–N stretching mode. The presence of an aliphatic chain was indicated by the C–H modes from 2970 to 2850 cm-1 and 1450–1380 cm − 1. A broad band around 3419 cm − 1 from the stretching mode of (O − H/N − H). These results strongly indicate that the biosurfactant contained aliphatic and peptide-like moieties.

#### Hydrogen and carbon nuclear magnetic resonance (NMR)

**Fig. 10:**
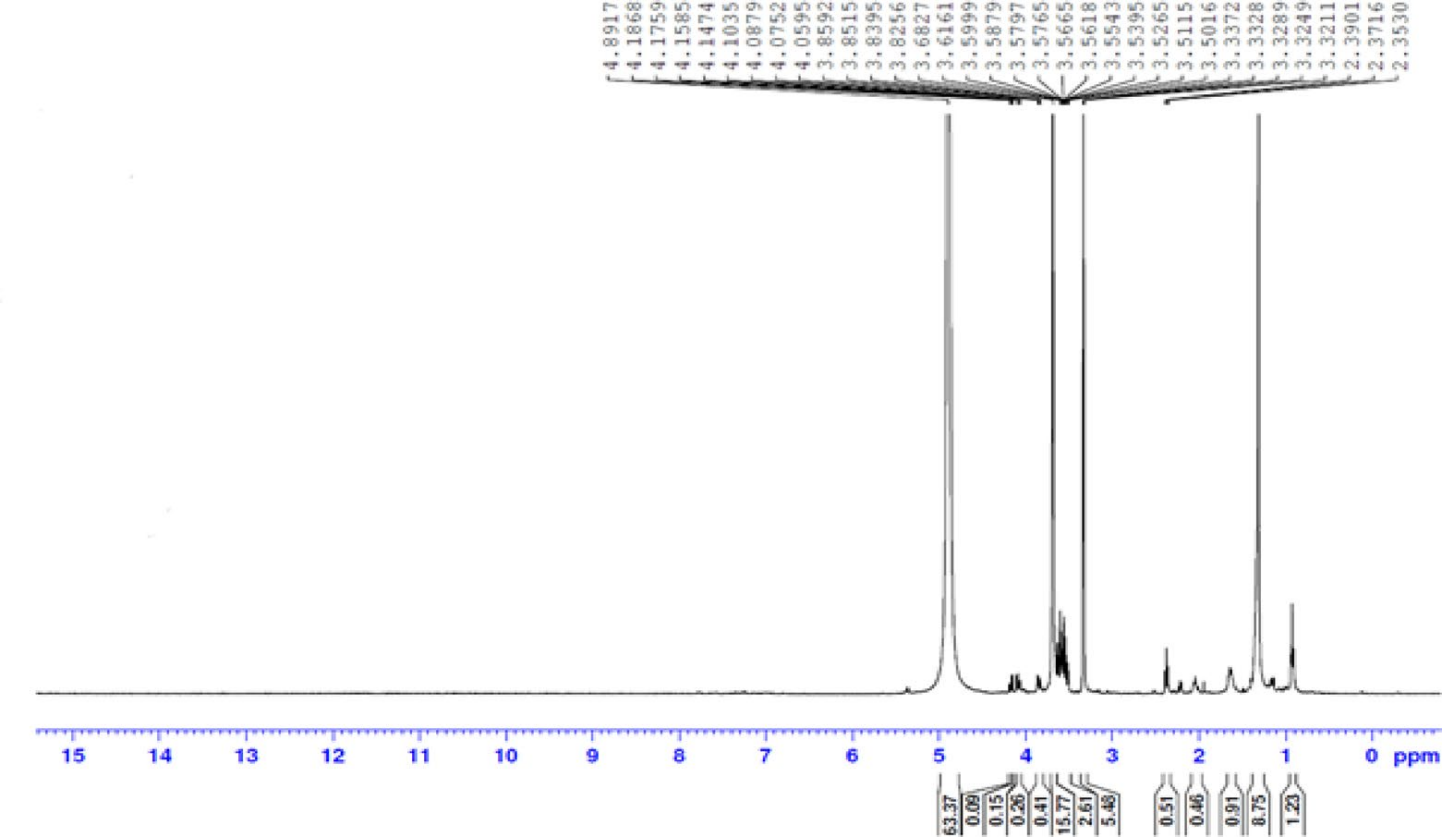
Purified surfactin from Bacillus subtilis 6633 in methanol-d¹: ¹H NMR spectrum. The signals are as follows: δ 0.91 ppm (methyl), δ 1.23 ppm (methylene proton envelope in hydrocarbon chain), δ 3.50–4.19 ppm (α-protons in cyclic heptapeptide), and δ ~ 4.89 ppm (β-methine proton in C3 hydroxy fatty acid chain). Amide N-H signals were eliminated by deuterium exchange. The spectrum is compatible with surfactin’s lipopeptide structure.

**¹**Methylene envelope (δ 1.23 ppm, hydrocarbon chain), terminal methyl (δ 0.91 ppm), α-protons multiplet (δ 3.50–4.19 ppm, cyclic heptapeptide), and β-methine proton (δ ~ 4.89 ppm, C3H hydroxy fatty acid)—a critical indicator—all demonstrated by ¹H NMR in methanol-d¹ confirmed the biosurfactant was surfactin as shown in [Fig. 10]. Due to deuterium exchange in the protic solvent, amide N-H signals are absent; the profile is consistent with published surfactin data. The biosurfactant’s pure surfactin structure was confirmed by ¹³C NMR (Table 1), which revealed all important resonances: aliphatic chain peaks (δ 29.05–13.04 ppm), β-methine carbon (δ 72.46 ppm, C3 hydroxy fatty acid), and eight carbonyl signals (δ 173.5–176.5 ppm; one lactone ester, seven peptide amides). Every piece of data completely matches surfactin.

**Table 1:** ¹³C NMR Spectral Data for the Purified Lipopeptide in CDCl₃ (¹³C NMR at 100 MHz).

δ (ppm)	Assignment	Group/Structural Feature
173.5	Ester Carbonyl	Lactone Ring (C = O)
174.2	Amide Carbonyl	Peptide Backbone (Glu1)
174.6	Amide Carbonyl	Peptide Backbone (Leu2)
175.1	Amide Carbonyl	Peptide Backbone (Leu3)
175.3	Amide Carbonyl	Peptide Backbone (Val4)
175.8	Amide Carbonyl	Peptide Backbone (Asp5)
176.2	Amide Carbonyl	Peptide Backbone (Leu6)
176.5	Amide Carbonyl	Peptide Backbone (Leu7)
69.75, 72.46	C3 (β-CH-OH)	β-Hydroxy Fatty Acid Chain
59.71, 61.26, 62.99	C-α	Amino Acid Backbone
46.98–48.26	C-α/Side Chains	Amino Acid Backbone/Side Chains
29.05–33.55	(CH₂)ₙ, β-CH₂	Aliphatic Fatty Acid Chain
13.04	Terminal CH₃	Fatty Acid Chain End Group

#### GC-mass analysis of beta-hydroxy fatty acids in surfactin

**Fig. 11:**
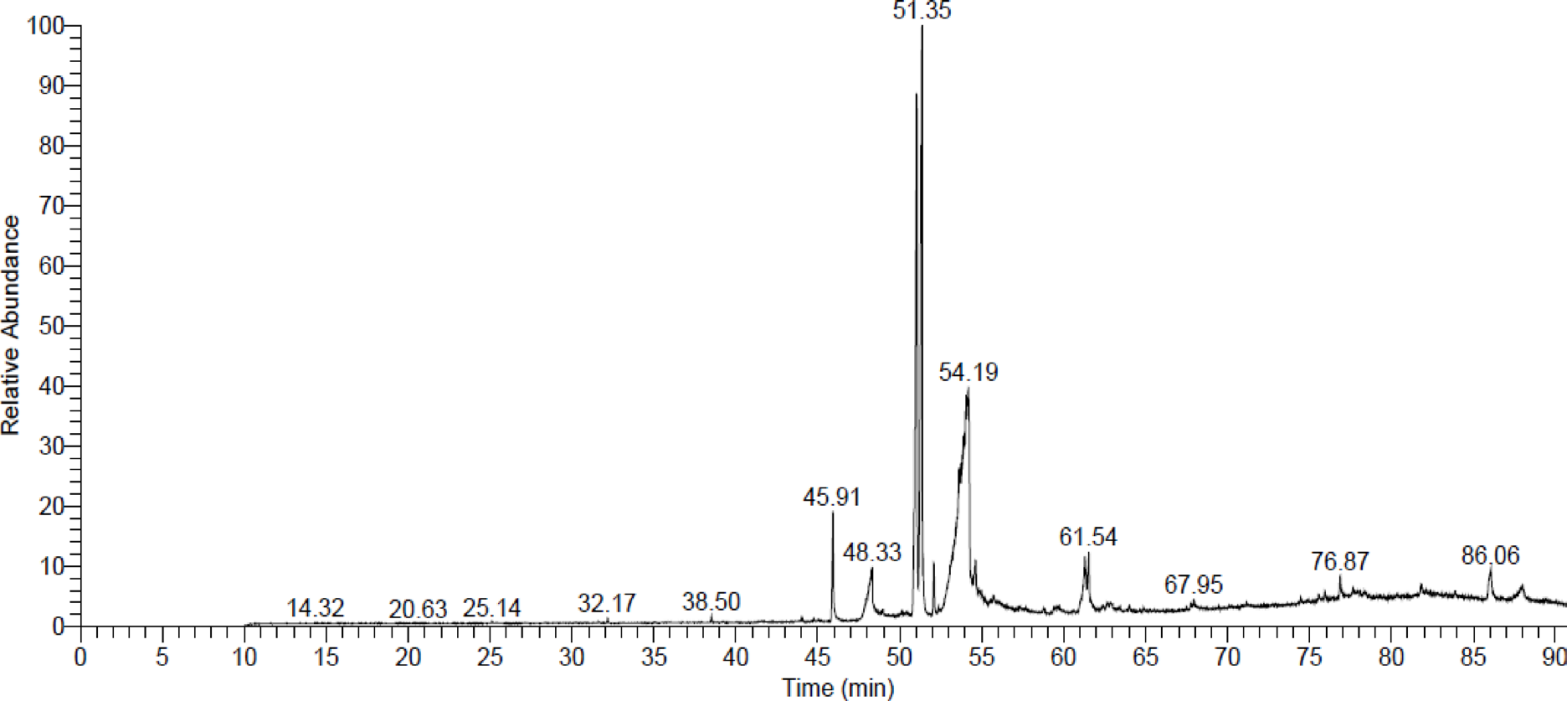
Purified surfactin from Bacillus subtilis 6633 was analyzed by GC-MS. Significant fatty acids, such as methyl oleate (27.42%, RT 51.34 min) and methyl linoleate (24.64%, RT 51.03 min), as well as 2,3-dihydroxypropyl esters of C18 fatty acids (RT 61.29 min), are present in the total ion chromatogram and extracted ion chromatogram at m/z 296. Relative abundance is displayed on the x-axis, and retention duration is displayed on the y-axis.

GC-MS analysis confirms that surfactin and its lipid precursors are present in the chromatographic profile, as seen in [Fig. 11]. The primary components of the complex chromatogram, methyl oleate (27.42%, RT 51.34 min) and methyl linoleate (24.64%, RT 51.03 min), were fatty acids and their derivatives, suggesting the existence of the C18 lipid variety frequently found in surfactin isoforms. More significantly, diagnostic pieces like 2,3-dihydroxypropyl esters of these particular C18 fatty acids, detected at RT 61.29 min, directly demonstrate the glycerol-fatty acid moiety, a characteristic of the surfactin structure. Ultimately, the coelution of these diagnostic pieces with the matching free fatty acid building blocks, which are consistently matched by the expected biosynthetic profile of surfactin produced by Bacillus subtilis 6633, supports the effective purification of a surfactin-rich extract.

### Preparation of the Dox-loaded surfactin/chitosan nanoparticles

#### Evaluation of Dox encapsulation efficiency and drug loading

Dox -Surf/CS and Dox - CS nanoparticles were successfully prepared using a micelle-assisted ionic gelation technique that combined TPP-induced ionic crosslinking of chitosan with surfactin’s capacity for micellar complexation. Encapsulation efficiency (EE %) and drug loading (DL %) were 90 ± 2% and 36 ± 2%, respectively, for Dox -Surf/CS nanoparticles and 74 ± 4% and 4 ± 1%, for Dox - CS nanoparticles, respectively, according to the following equation: y = 18.16x + 0.0123.

#### In vitro release study of Dox from both the DOX-Surf/CS and DOX- CS nanoparticles

**Fig. 12:**
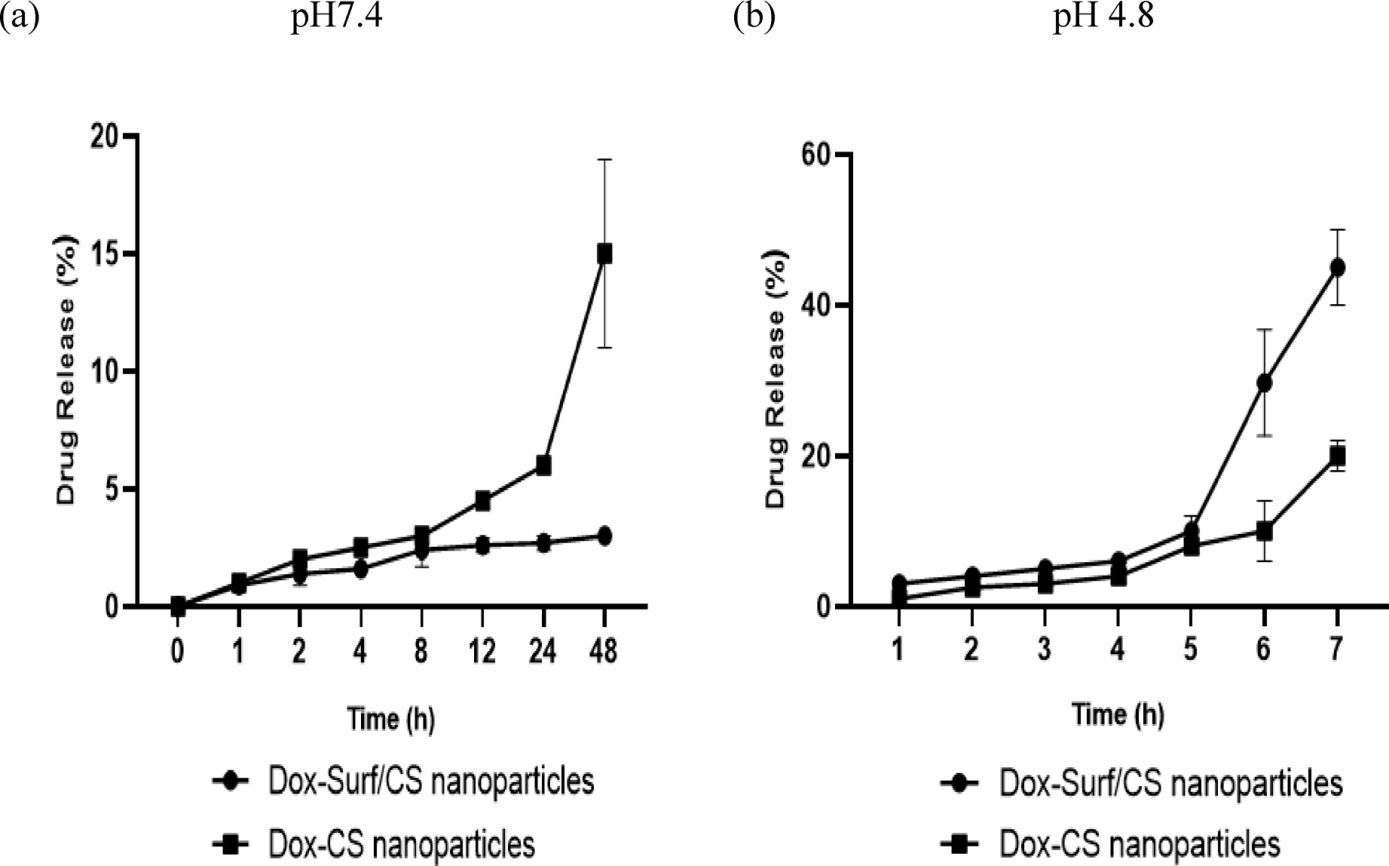
Dox release profiles from Dox-Surf/CS and Dox-CS nanoparticles at pH 4.8 and 7.4 in vitro. Dox is released slowly from Dox-Surf/CS and Dox-CS nanoparticles at pH 7.4, at roughly 3 and 15%, respectively. However, the release of Dox from Dox-Surf/CS and Dox-CS nanoparticles is higher in an acidic environment at pH 4.8, at roughly 45 and 20%, respectively. The findings are displayed as mean ± SD (*n* = 3, *p* < 0.0001).

The Dox release profile exhibited a single-phase release [Fig. 12-a], with 45% released from Dox-Surf/CS nanoparticles versus 20% from CS nanoparticles after 48 h at pH 4.8; the difference was statistically highly significant (*p* < 0.0001). Whereas [Fig. 12-b], the rate of Dox release at pH 7.4 (normal pH) from Dox-Surf/CS nanoparticles and CS nanoparticles was approximately 3% and 15%, respectively, over 48 h, which is notably slower than that at the cancer pH with significant (*p* < 0.0001).

#### Morphology of Dox‑Surf/CS nanoparticles

**Fig. 13:**
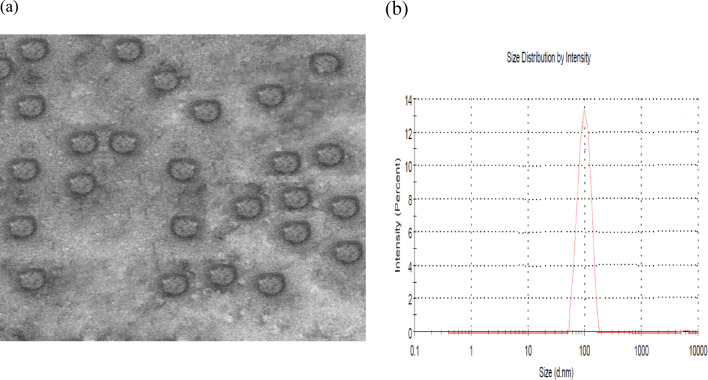
Dox-Surf/CS nanoparticle morphological and size characterization. (**a**) TEM picture of spherical nanoparticles made by ionic gelation with micelle assistance. (**b**) Dynamic light scattering (DLS) study results show a limited size distribution of monodisperse nanoparticles with an average size of 100 ± 2 nm, zeta potential of + 3 ± 0.5 mV, and PDI of 0.019 ± 0.01.

The TEM image revealed that Dox-Surf/CS nanoparticles prepared by the micelle-assisted ionic gelation technique were spherical in shape, as shown in [Fig. 13-a]. The particle size and PDI are illustrated in [Fig. 13-b]. The average size of nanoparticles determined by DLS was 100 ± 2 nm, the zeta potential was + 3 ± 0.5 mV, and PDI was 0.019 ± 0.01 with a narrow monodispersed unimodal size distribution pattern.

### In vitro studies of Dox-Surf/CS nanoparticles

#### Hemolytic activity assay

**Fig. 14:**
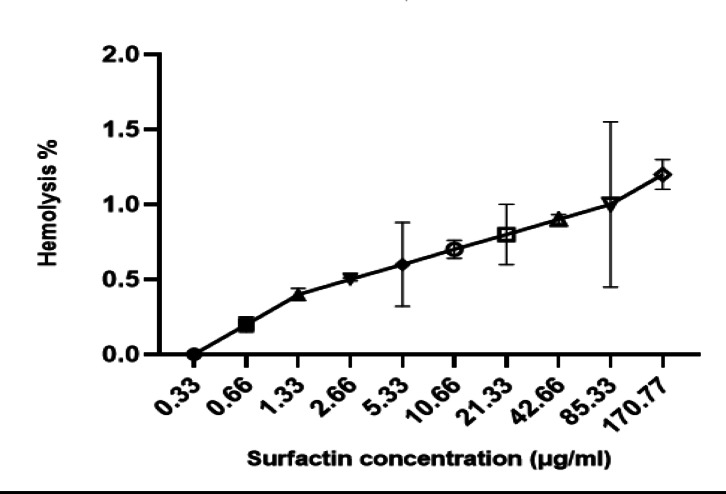
Surfactin’s hemolytic activity against red blood cells at doses between 0.33 and 170.66 µg/mL. The highest hemolysis level was 1.2%, and it progressively decreased from 0.33 µg/mL to 0 µg/mL, at which point no hemolysis was observed. The findings are statistically significant (*p* < 0.0001). Acid precipitation and extraction using 70% ethanol were used to extract and purify surfactin.

The hemolytic activity of surfactin was measured using two different techniques, with concentrations ranging from 0.33 to 170.66 µg/mL. As shown in [Fig. 14], The greatest hemolysis for surfactin was 1.2% at 170.66 µg/mL, and it gradually decreased to 0% at 0.33 µg/mL with significant (*p* < 0.0001).

#### In vitro cytotoxicity on normal and cancer cell lines

The assays were carried out based on the calculation of the drug loading capacity of Dox inside Surf/CS and CS nanoparticles Table 2.

**Table 2:** The concentrations of free Dox, Dox-Surf/CS, and Dox/CS nanoparticles used in the In Vitro cytotoxicity and anticancer assays.

Conc of free Dox used as positive control (µg/ml)	Equivalent Dox conc. inside Surf/CS nanoparticles (µg/ml)	Equivalent Dox conc. Inside CS nanoparticles without Surf (µg/ml)
25	70	700
12.5	35	350
6.25	17.5	175
2.125	8.75	87.5
1.56	4.375	43.75
0	0	0

**Fig. 15:**
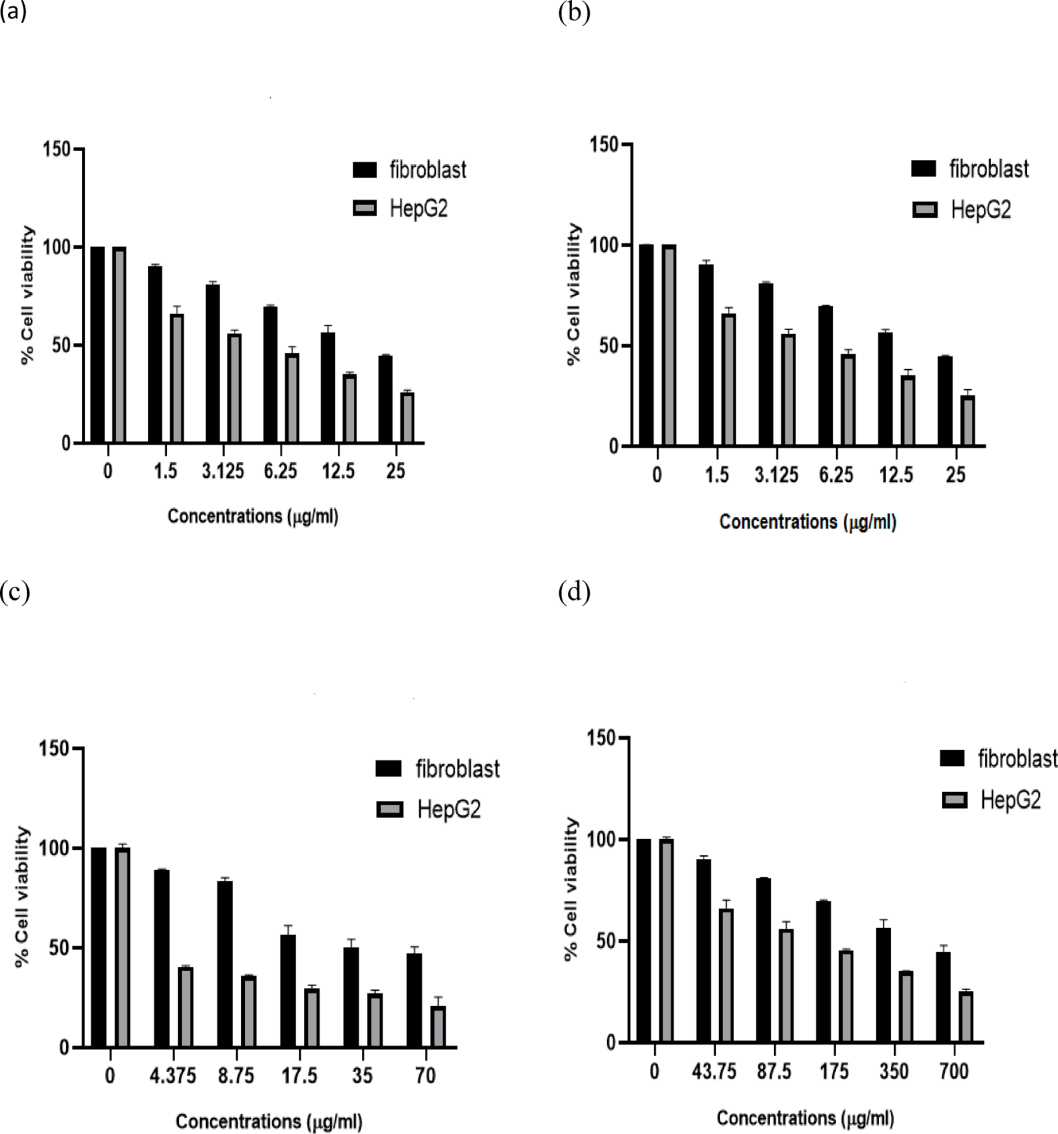
The in vitro cytotoxicity of (**a**): free doxorubicin (Dox), (**b**): free surfactin (Surf), (**c**): Dox-Surf/CS nanoparticles, and (**d**): Dox-CS nanoparticles against HepG2 cancer cells and normal fibroblasts (FB). Dox-Surf/CS nanoparticles have demonstrated selective cytotoxicity, drastically reducing HepG2 cell viability in comparison to normal cells. Significant differences between FB and HepG2 cells were seen in all formulations (*p* < 0.0001). The data are mean ± SD.

**Fig. 16:**
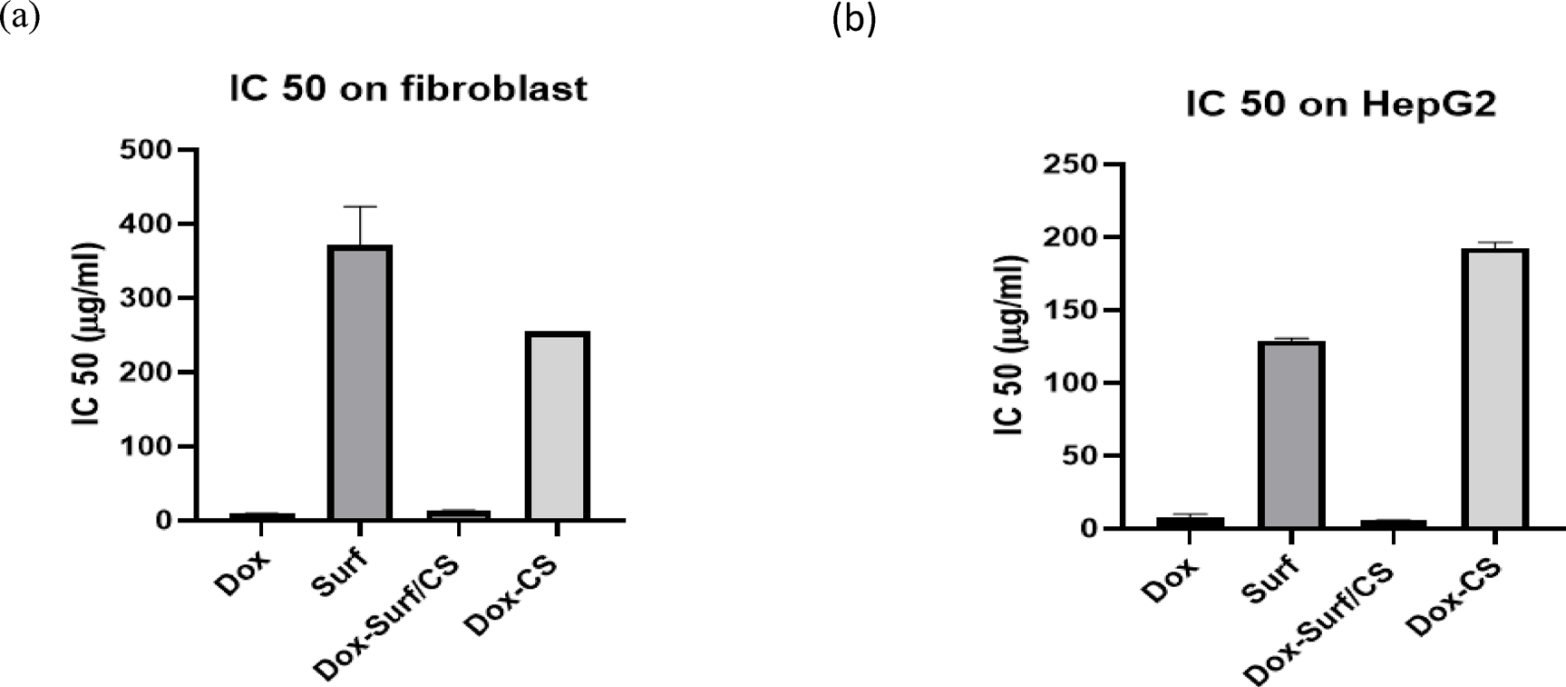
IC_50_ values for free Dox, free surfactin, Dox-Surf/CS, and Dox-CS nanoparticles are compared against (**a**) FB and (**b**) HepG2 cells. When compared to other formulations, Dox-Surf/CS nanoparticles had the lowest IC_50_ value in HepG2 cells (5.40 ± 0.1 µg/mL, more powerful) and the highest IC_50_ value in FB cells (12.78 ± 0.47 µg/mL, less hazardous) (*p* < 0.0001).

Results from [Fig. 15-a] indicate that after 24 h treatment with free Dox (25–0 µg/ml), normal fibroblast (FB) cells had viability of 44.98–100% compared to 25.16–100% in HepG2 cells, which was significantly lower (*p* < 0.0001). The empty CS nanoparticles (1000–0 µg/ml) gave viability of approximately 97–100% in FB cells, which was significantly lower compared to 95–100% in HepG2 cells (*p* < 0.0001). Free Surf (1000–0 µg/ml) gave viability of 45.66–100% in FB cells, which was significantly lower compared to 38.48–100% in HepG2 cells (*p* < 0.0001) [Fig. 15 -b]. Dox-Surf/CS nanoparticles (70–0 µg/ml, equivalent Dox; Table 1) gave viability of 47.21–100% in FB cells, which was significantly lower compared to 22.86–100% in HepG2 cells (*p* < 0.0001) [Fig. 15 -c]; Dox/CS nanoparticles (700–0 µg/ml, equivalent Dox; Table 1) gave viability of 76.52–100% in FB cells, which was significantly lower compared to 68.48–100% in HepG2 cells (*p* < 0.0001) [Fig. 15-d]. Multiple t-test confirmed high significance between FB and HepG2 cells across all treatments (*p* < 0.0001). IC_50_ values for normal fibroblasts [Fig. 16-a], Dox-Surf/CS nanoparticles had an IC_50_ value of 12.78 ± 0.47 µg/ml, which was significantly higher (less toxic) compared to other formulations. In contrast, with HepG2 cancer cells [Fig. 16-b], the Dox-Surf/CS nanoparticles were the most potent, with an IC₅₀ value of 5.40 ± 0.1 µg/ml, which was more effective compared with free Dox with an IC₅₀ value of 7.68 ± 0.08 µg/ml (*p* < 0.0001).

## Discussion

A comprehensive pipeline established surfactin synthesis, purification, and characterization from *Bacillus subtilis*6633, marking its first use as a doxorubicin (Dox) nanocarrier. Firstly, PCR amplification of a 176 bp srf gene segment at 62 °C annealing confirmed non-ribosomal synthesis capability, aligning with Guo et al.^36^. In order to optimize surfactin synthesis, the current work employed a one-factor-at-a-time (OFAT) methodology, methodically improving each variable separately while keeping the others constant. This method made it simple to observe how each variable affected the surfactin yield. It was particularly useful for understanding the process and for early optimization. While statistical design techniques such as Response Surface Methodology (RSM) are beneficial for determining the interdependence of variables, OFAT remains a popular and practical approach for small-scale optimization and early-stage research^37,38^. As a result, the selected approach was appropriate for the current work’s scope, which focused on creating baseline optimum circumstances before future multivariate optimization investigations. The present study creates a fundamental optimal framework that could be improved in future research using multivariate statistical techniques.

Optimized mineral salt medium (2% glucose, 0.4% ammonium sulfate, pH 6.7, 35 °C) yielded 3 g/L surfactin—far superior to prior *B. subtilis*6633 reports: 0.18–0.29 g/L by Cagri-Mehmetoglu et al.^39^, < 1 g/L by de Sousa et al.^40^, and 0.8 g/L by Dehghan-Noudeh et al.^41^ while high glucose/ammonium sulfate suppressed yields via catabolite repression, consistent with Umar et al.^25^. According to^22,36,42,43^, surfactin production can range from 2 to more than 5 g per liter of fermentation broth in optimally developed fermentation processes. The production attained in this study (3 mg/ml, or 3 g per liter) is within the previously reported production range. Furthermore, it has been demonstrated that surfactin production varies greatly and depends on the strain’s metabolic capacity and growing environment^44^.

To obtain a high-purity product, multistep chromatography was achieved through a desalting technique to eliminate contaminants, followed by an anion-exchange (HiTrap Q Sepharose, ANX, DEAE-cellulose) at pH 6.8 to exploit anionic Asp/Glu residues, resulting in a sharp eluted peak that was further confirmed by HPLC^4^. In addition, TLC results showed Rf 0.54, which matches TLC Rf 0.53 by Chandran and Prabhakaran^45^ and TLC Rf 0.56 by Kumar et al.^46^. Results of FTIR displayed characteristic amide I (~ 1650 cm⁻¹), amide II (~ 1540 cm⁻¹), C-H stretches (2970–2850 cm⁻¹), and lactone carbonyl (~ 1730 cm⁻¹), which in consistent with Meena et al.^47^, and De Faria et al.^48^. In addition, ¹H NMR showed aliphatic (1.25–1.55 ppm), peptide (7.2–8.0 ppm), β-hydroxy (5.06 ppm), and methoxy (3.67 ppm) shifts; ¹³C NMR confirmed lactone carbonyl, peptide carbons, and methoxy (~ 49 ppm) of methylated surfactin. Final confirmation using GC-MS revealed dominant methyl oleate (27.42%) and linoleate (24.64%) C18 isoforms, consistent with Qin et al.^49^, and Bochynek et al.^50^.

The ideal anticancer drug delivery system (DDS) provides prolonged, targeted release of drugs, enhancing efficacy while minimizing systemic side effects. Such systems protect drugs from rapid metabolism and clearance^51,52^. Surfactin, an amphiphilic biosurfactant, self-assembles into micelles that effectively encapsulate hydrophobic drugs, improving their solubility, stability, and bioavailability^11,53^. This study innovatively developed a surfactin-chitosan hybrid nanoparticle DDS via micelle-assisted ionic gelation. The surfactin core greatly enhanced the encapsulation of doxorubicin (Dox), achieving 90 ± 2% encapsulation efficiency and 36 ± 2% drug loading, far exceeding plain chitosan nanoparticles (74 ± 4% and 4 ± 1%, respectively) due to superior compatibility between surfactin’s hydrophobic chain and the drug^11,54,55^. Physicochemical characterization confirmed successful synthesis. TEM showed distinct spherical nanoparticles, and DLS indicated a monodisperse population with a mean hydrodynamic diameter of 100 nm and a very low PDI of 0.019. This small, uniform size is critical for exploiting the Enhanced Permeability and Retention (EPR) effect for tumor targeting^56^. Zeta potential was slightly positive (+ 3 mV), suggesting the chitosan shield protects the anionic surfactin micelles. This charge may improve colloidal stability, circulation time, and interaction with negatively charged cancer cell membranes^31,57^. Our hybrid nanoparticles showed advantages compared to other systems over surfactin-only as nanocarriers^11,58^ and higher loading capacity than CaCO₃-based nanoparticles^59^. The integration of surfactin was key to transforming the drug release profile and therapeutic efficacy of the nanocarrier. While conventional chitosan-only nanoparticles (Dox/CS) exhibit slow, diffusion-controlled release with poor pH sensitivity (20% at pH 4.8 vs. 15% at pH 7.4), surfactin enables a core-shell structure. This adds a strong pH-responsive mechanism: in the acidic tumor microenvironment, protonation of chitosan’s amine groups weakens ionic crosslinking, destabilizing the micelle core and swelling the shell. This dual rupture causes a rapid, triggered release in Dox-Surf/CS nanoparticles (45% at pH 4.8 vs. only 3% at pH 7.4)—a 15-fold differential driven by surfactin that enhances tumor selectivity^60,61^. Cytotoxicity assays revealed the system’s enhanced selectivity, where free Dox was toxic to both normal fibroblasts and HepG2 cancer cells. However, Dox/CS nanoparticles reduced toxicity but also had a weaker anticancer effect. In contrast, Dox-Surf/CS nanoparticles showed the highest cytotoxicity against HepG2 cells with significantly less toxicity to normal cells. This selective toxicity is attributed to surfactin’s amphiphilic nature, which improves cellular uptake in cancer cells and enables pH-triggered release, bypassing P-glycoprotein efflux mechanisms^62,63^.

## Conclusion

The present study has thoroughly and reliably investigated the characterization and systematic optimization of producing bioactive surfactin lipopeptide from *Bacillus subtilis* 6633. Our work has progressed with the construction and development of the DOX-Surf/CS nanoparticle. When directly compared with many studies, our approach showed obvious advantages in drug loading capacity, remarkable release kinetics, and strong selective cytotoxicity. In addition, the constructed nanocapsules revealed less cytotoxicity on normal cells compared to free DOX. These results emphasize the potential of surfactin as a promising nanocarrier.

## Limitations and future perspectives

Despite the promising findings, this study has some limitations. The present study was mostly restricted to in vitro evaluation; hence, in vivo safety, pharmacokinetics, biodistribution, and therapeutic efficacy of the developed formulation have to be established. Furthermore, the detailed molecular mechanisms responsible for the increased cytotoxic activity need to be clarified. Future studies need to evaluate DOX-Surf/CS nanoparticles in tumor-bearing animal models to determine their tumor targeting ability, systemic toxicity, and therapeutic efficacy compared with free doxorubicin. Further optimization of the platform and investigation of its applicability for the delivery of other hydrophobic anticancer agents will extend its potential utility in nanomedicine and support the future translation of microbial biosurfactant-based drug delivery systems into clinical applications.

## Data Availability

All data generated or analysed during this study are included in this article and its supplementary information files.
